# 

**Published:** 1877-07

**Authors:** 


*CONTENTS.*

| | Page |
|---|---|
| Parental Corrections . . | 181 |
| Health Rules . . . . . . . | 188 |
| Healthy Bread . . . . . . | 188 |
| Child Murder . . . . . . . | 191 |
| Cheapest Food . . . . . . | 191 |
| The Dollar and Blood Aristocracy . . . . . . . | 191 |
| Early Marriages . . . . | 194 |
| Fruits in Summer . . . . | 195 |
| Count Golfalioneri . . . | 197 |
| Care of the Eyes . . . . | 197 |
| To Prevent Sleeping in Church . . . . . . . | 198 |
| Damp Walls . . . . . . . | 199 |
| Sores . . . . . . . . . . . | 200 |
| Heat Disease . . . . . . . | 201 |
| Sleeping Together . . . . | 202 |
| Rancid Butter . . . . . | 203 |
| A Surfeit . . . . . . . . | 204 |
| Spring Diseases . . . . . | 204 |
| Cure for Drunkenness . . | 206 |
| The Eyes . . . . . . . . | 206 |

